# Sociological perspectives of social media, rumors, and attacks on minorities: Evidence from Bangladesh

**DOI:** 10.3389/fsoc.2023.1067726

**Published:** 2023-02-24

**Authors:** Sajal Roy, Ashish Kumar Singh, Kamruzzaman

**Affiliations:** ^1^University of New South Wales, Sydney, NSW, Australia; ^2^School of Politics and Governance, National Research University Higher School of Economics, Moscow, Russia; ^3^Begum Rokeya University, Rangpur, Bangladesh

**Keywords:** social media, religious extremism, violence, minority, Bangladesh

## Abstract

Social media platforms serve as essential modes of communication for billions of people around the world. They host a diverse range of content—from the personal and social to the political serving as an important channel for connecting people for spreading ideas. However, given their widespread penetration into everyday social and political life, they have become a tool for spreading rumors and disinformation that may often misrepresent or distort reality and have in many cases incited violence. In Bangladesh, perpetrators over the last decade have utilized social media platforms to spread rumors and to mobilize mobs to launch violent attacks on minority groups. Drawing from social movement theories on how the nexus between social media and political violence works, this paper examines a sample of five cases during the periods of 2011–2022. We exemplify minority attacks being instigated by social media rumors to understand their nature and causes thereof. The study finds that religious extremism, the absence of legal safeguards, and the culture of impunity are the primary triggers, to a varying degree, for the social media rumor-instigated attacks on minorities in Bangladesh.

## 1. Introduction

This study argues that the main causes of social-media-rumor-instigated violent attacks on religious minorities in Bangladesh are an increasing religious extremism, the lack of legal safeguards for minorities, and the culture of impunity. These findings are based on a detailed content analysis of five cases between 2011 and 2022. Social media has evolved into an important mode of communication. It is fuelled by a diverse range of content from the personal to the political. It's used by 58.4% of the world's population, for an average of 2 and a half hours/day (Chaffey, 2022). The most popular social media platforms are Facebook, YouTube, WhatsApp, Instagram, WeChat, and Twitter, but there are also far darker, far less censored “boards” such as Redditt, Telegram, 4/8 chan and the like. These platforms are widely used for the dissemination and consumption of information (Ahmad et al., 2018). Nowadays, social media platforms serve as an important channel for connecting people, spreading ideas, and receiving feedback (Bhanot, 2012). In Bangladesh 20.5% of the population live below the national poverty line (Asian Development Bank, 2019) and rumors are spreading very quickly due to the easy access to social media across the country, with about 22% of its population using social media (Kemp, 2020).

According to the 2022 report on the mobile economy released by Global System for Mobile Communications Association shows increasing use of the internet and smart phones in Bangladesh. In March 2022, it included 11 million using broadband and 113.9 million with smart phones (GSMA, 2022).

There were 36 million social media users in the country as of 2020, most of whom use Facebook (Kemp, 2020). Attacks on religious minorities in Muslim-majority Bangladesh have increased in recent years, with many such incidents the result of rumors or fake posts published on social media. Since 2010, misuse of social media has frequently posed challenges to Bangladesh's internal peace and security. An alarming number of attacks against minority communities were initiated by defaming posts and related rumors on social media, beginning with the incidents in Ramu (2012), leading to Pabna (2013), Comilla (2014), Rangpur (2017), Bhola (2019), and Comilla (2021).

The development of social networking sites and digital news outlets has exacerbated the spread of rumors and untruths. This study mainly considers five examples covering attacks on religious minorities in Bangladesh during the last decade. These are purposively chosen depicting our authorial biasness to achieve the study aim. We have offered our reflections drawing insights from Newman et al. (2018) as well as Meel and Vishwakarma (2020).

This study is designed to better understand the trends and causes of social media rumors in Bangladesh. The organization of this paper is as follows. The first section examines the growing number of internet and social media users in Bangladesh. Then, the paper investigates both the trends and consequences of attacks on minorities in Bangladesh over the last decade. Finally, we explore the actual cases of attacks on minorities due to the spread of rumors and “fake news” on Bangladeshi social media.

## 2. Linking social media and rumors: Brief reflections from literature

Social media platforms have provided immediate access to and dissemination of opinions, ideas and information. As an entity, social media has developed into a platform for discussions, ideology expressions, information sharing, and emotion as well as a feeling exchange (Meel and Vishwakarma, 2020). However, the veracity of content shared on social media platforms has come under question (Newman et al., 2018). Unlike traditional media, social media posts are rarely subject to fact checking (Global Digital Report, 2019). Out of the world's 7.676 billion people, 4.388 billion access the internet, with 3.484 billion using social media. Nearly half of all people on earth rely on the internet for information (Newman et al., 2018). Meel and Vishwakarma (2020) demonstrate the ways in which social media offers a platform for rapidly generating and sharing data. Their study provides a classification of false information that includes the following 10 categories: rumors, fake news, misinformation, disinformation, clickbait, hoaxes, satire/parody, opinion spam, propaganda, and conspiracy theories. Meel and Vishwakarma (2020) categories the false information but they do not clearly define what they mean by rumors and fake news. In this study we clearly define what is the meaning of fake news and rumors extending from the work of Meel and Vishwakarma (2020).

False information claims are rumors, whereas correct information is not (Seo et al., 2012). Rumor is “a proposition of belief that is officially unverified when it is issued and should deal with either current events or topical issues to express the emotional needs of the community and/or to help people make sense [of it] in the context of ambiguity, danger, or potential threat” (Kim et al., 2019, p. 593). Such situations usually arise due to limited knowledge; when there is a psychological demand for understanding; or when there is a threat (DiFonzo and Bordia, 2007).

A rumor can be a hoax, a joke, a short story, or a lack of information (Lewandowsky et al., 2012). It can be both “breaking news” and [yet possess] insufficient proof or supporting evidence (Turenne, 2018). The study of Derczynski et al. (2015) offers a more comprehensive typology of rumors. For them, rumors are classified into four types: speculation, controversy, misinformation, and disinformation. Inaccurate information is typically the result of unintentional knowledge gaps, while misinformation is frequently intentional and includes defamatory statements, insults, and practical jokes. Disinformation, also referred to as false information, is produced and disseminated with the *intention* of misleading people, as opposed to misinformation (Al-Zaman, 2019). Because they share several traits, [the terms] “rumors,” “misinformation,” “disinformation,” and “fake news” are commonly used interchangeably (Tandoc et al., 2020). For instance, while some studies define fake news as disinformation (Tandoc et al., 2020), others call it misinformation (Tandoc et al., 2020). False news/information can thus be both intentional and unintentional. So-called “fake news” and rumors are two closely related components in the information ecology (Duffy et al., 2019). In this study, we prefer to use rumor to avoid conceptual ambiguity.

## 3. Unpacking social movement theory

Social media has recently contributed to the development of a new public sphere and space for activism in Bangladesh. For instance, the Shahbag movement contributed to a larger scale and greater intensity of “social movement's social media and political action” in social media and everyday life (Chowdhury, 2018). Shahbag movement left numerous traces in Bangladeshi society, politics, culture, and social media, according to a study that examined the movement's role in social media and examined related micro narratives (Chowdhury, 2018). It is a space for social and political discourse that is still actively creating new actions and meanings today.

We unpack the social movement theory to offer three different types of larger causal mechanisms to ways in which social media have been influencing political violence over the past few decades in Bangladesh. Social movement theory focuses on the degree and type of motivation that influences people's decisions to participate in politics. Scholars (such as Bouhana and Wikström, 2010; Stekelenburg and Klandermans, 2010) suggest that explanations of political violence must also consider how people deal with the moral aspect of their actions, or how violence is justified.

Although the larger discursive environment may provide individuals and groups with a general sense of legitimacy and drive, it is often the interaction with others that provides the social and emotional push for action. Social media, in addition to serving as a discursive environment and a channel for social connection also provide the practical knowledge needed to turn intention into action. The study of Wahlström and Törnberg (2021) identifies three causal mechanisms to understand how political violence is influenced by social media.

### 3.1. Discursive opportunity structure

Given the significant increase in the number of people who use social media platforms as their primary news source, the key discourse (s) on those platforms is regarded as important aspects of the broader national DOS, distributing ideas that may justify violent action. The creation and spread of online misinformation, rumors and arguments can be understood by the same logic.

### 3.2. Trans-local group dynamics

Trans-local group dynamics that were formerly limited to local face-to-face interactions are now able to occur trans-locally *via* online social media. If discursive opportunities are primarily about the nature of the ideas existing in a media environment, “trans-local group dynamics” refers to interactions that occur within polarized groups that legitimize, create resonance for, and further improve user opinions. Participants do not only discuss abstract ideas and viewpoints, but also provide feedback, mutual recognition, and emotional responses that encourage action. When associated with an increase in religious fundamentalism, rumors can easily result in collective action.

### 3.3. Practical information and coordination of collective activities

Not only does a social medium play a part in deciding the occurrence of militant operations, but it also plays a role in identifying their specific objectives and methods of operation. In general, this particular social media technique has received less attention in the social movement literature.

## 4. Interconnection between social media and violence

Reports show that the use of social media and violence against minorities are linked. Every year, religious minorities in Bangladesh are attacked after false claims are spread on the internet. The trend is as follows: rumors circulate within a local group that persons of a minority group have defamed Islam, and this “false news” quickly travels online to encourage violence against such minorities (Hansan, 2021). Although social platforms are useful media for promoting hatred and violence against minorities in Bangladesh, lethal rumors attract crowds who ultimately assault minority members for supposed blasphemy (Hansan, 2021).

Facebook is the most popular social network in Bangladesh with people using it for communication, social networking and other objectives (Morshed et al., 2017). Facebook has also enabled the worst forms of financial fraud, extremism and violence. Recent events have exacerbated the problem (Minar and Naher, 2018). Ramu, in 2012, was the site of the first significant occurrence of ethnic violence, followed by Pabna in 2013, Comila in 2014, Rangpur in 2017, and Comilla again in 2021. Each event demonstrated the effects of rumors/fake news resulting in arson and violence.

## 5. Recent incidences of “fake news” and rumors about minorities on social media

In the last decade, against minorities have taken place in Bangladesh, some of which were clearly connected to false information posted on social media. In this section, we offer following examples.

### 5.1. Example 1

In Ramu, Cox's Bazar, Buddhist temples were burnt down in September 2012. An unknown/fake Facebook user tagged a local Buddhist, Uttam Kumar Barua, on an image of a burning Quran. Angry with this post, a Buddhist temple was set on fire and an attack was launched against the Buddhist community (bdnews24.com, 2012a; The Daily Star, 2012). Soon after, the violence spread to Ukhiya Upzilla in Cox's Bazar and Patiya Upzilla in Chittagong district. The attackers targeted not only the Buddhist sites but also Sikh Gurudwaras and Hindu temples (bdnews24.com, 2012b).

### 5.2. Example 2

In the Bonogram neighborhood of Pabna district, a mob went on the rampage on November 3, 2013. Copies of a Facebook page were distributed alleging a Hindu named Rajib Saha (student of class X) for supposedly defaming the Prophet Mohammad. Without any attempt to seek out the truth or check the facts, an angry mob from the majority ethnic group vandalized more than twenty-five Hindu homes, destroyed several Hindu temples and forced nearly one hundred and fifty families to flee the region (The Daily Star, 2013).

### 5.3. Example 3

In April 2014, fake news spread in the HomnaUpzilla of Comila district that Prophet Muhammad had been defamed in Facebook posts by some Hindus (The Daily Star, 2014). The angry Muslim community members in their hundreds assaulted the temples and homes of at least twenty-eight Hindu households. Before the attacks, pamphlets claiming that two Hindu youngsters wrote insulting statements about the prophet on Facebook were distributed by madrasas (Bangladeshchronicle.net, 2014).

### 5.4. Example 4

A Facebook account with a username of a Hindu, Titu Roy, slandered the Prophet Muhammad. As a result, a clash broke out in Thakurpara, Rangpur on November 10, 2017. One person was killed, and twenty others were injured including seven police officers (The Daily Star, 2017a). Upon inquiry, the police found that the Facebook ID used to supposedly spread hatred against Muslims was created using an untraceable mobile phone number, which was then deactivated; the police never discovered its owner (The Daily Star, 2017b).

### 5.5. Example 5

On the morning of October 13, 2021, claims of defamation of the Muslim holy book Quran surfaced from a makeshift temple in Comilla district, after reports of finding a copy of the Quran on the lap of an idol spread on social media sites. During the Durga Puja celebration, Muslim mobs sparked communal violence against Hindu communities across Bangladesh from October 13 to October 19, 2021. Over 120 temples and improvised worship sites were vandalized (The Guardian, 2021). As of October 22, 2021, at least ten people had been slain across the country, including seven Hindus, in the “worst sectarian violence this year” (bdnews.com, 2021). Overall, our examples clearly show the complex nexus of social media, rumors, and violence against minorities in Bangladesh. The figures below show the frequency, severity, intensity and types of damage caused by these acts.

**Figure 1:**
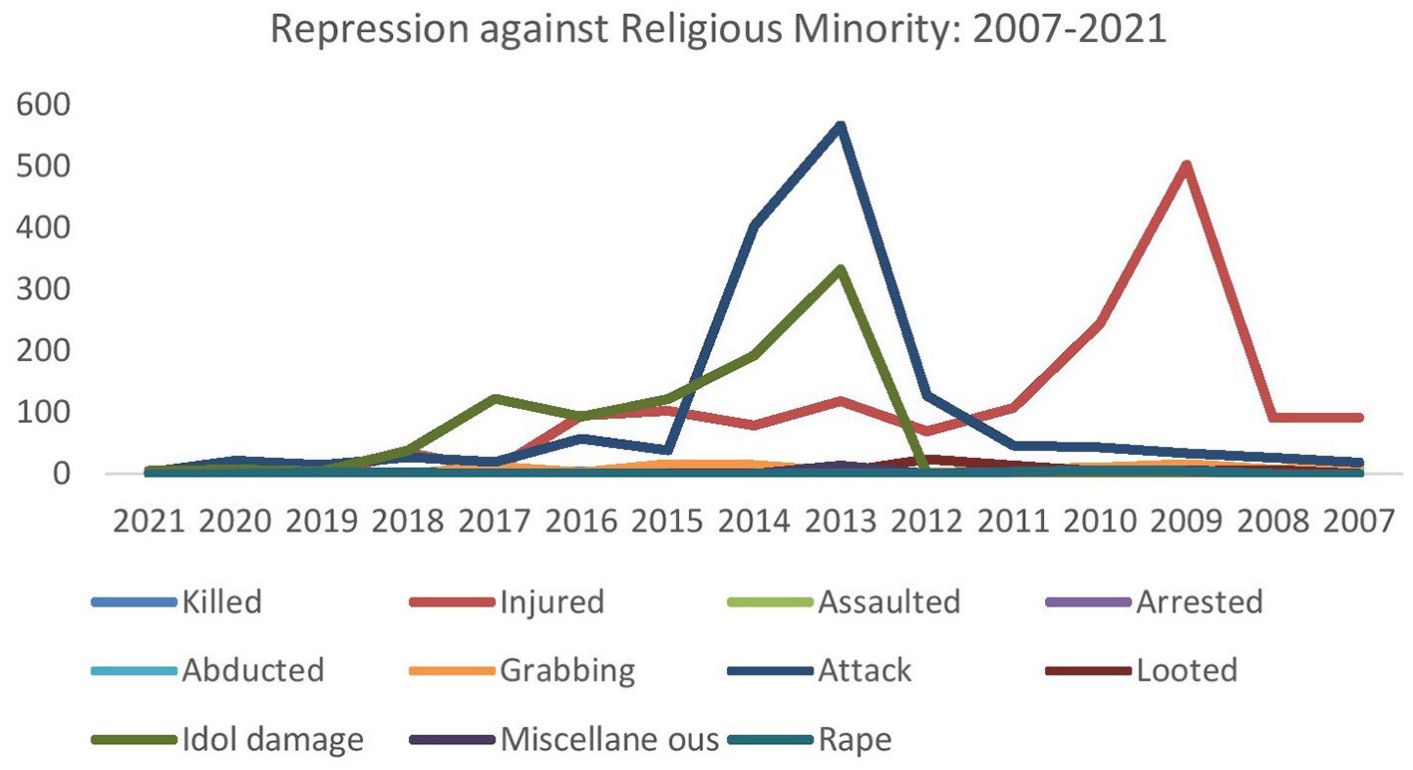
Repression against religious minorities in Bangladesh from 2007–2021.

Figure 1 shows how minorities have been victims of violence in various ways between 2007 and 2020. They have been either killed, raped, injured, assaulted, arrested, or abducted at various times. They are also victims of grabbing, looting and frequent idol damage. According to the Odhikar report, 20 minority people were killed, 1,538 people were injured, and 21 people were raped in attacks on minorities attack in Bangladesh between 2007 and 2020. On hundred and seven families lost land and homes during this time. Three hundred and ninety-seven temples were damaged, and 1,043 families lost their property (Odhikar, 2021).

**Figure 2:**
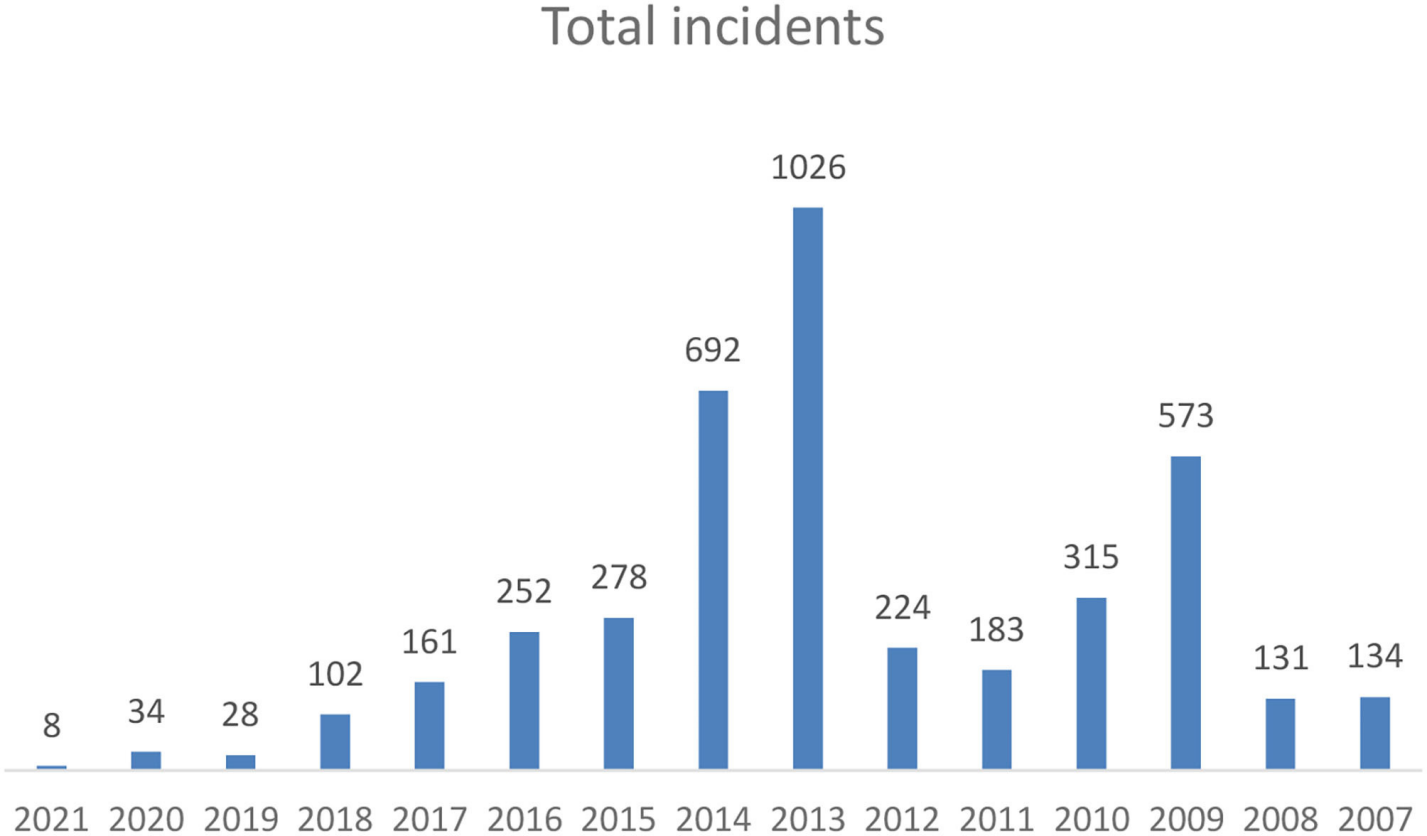
Total number of incidents per year.

According to Odikar's most recent report, the incidence of minority attacks in Bangladesh has been increasing. The total number of known minority attacks was 134 in 2007, but increased dramatically one year later and by 2009, reached 573 (Figure 2).

## 6. Discussion of findings: Identified causes of attacks on minorities

### 6.1. Connection of theoretical framework with discussion

The theoretical foundations of social movement theory provide an overview of the connections between social media and violence against minorities. Using discursive opportunity structure (DOS) pre-planner, fundamentalists disseminate online rumors, arguments, and misinformation to incite large numbers of people to take violent action against minorities. Contrarily, trans-local group dynamics offer feedback, mutual acknowledgment, and emotional reactions that motivate action in addition to discussing abstract concepts and points of view. Even though there is a lack of useful information and coordination of group activities related to social media, it has not received much attention in the literature on social movements. Because of this, communal violence continues to receive less attention as a violation of human rights. In addition, a number of factors, such as the lack of penalties for violence against minorities, lack of legal safeguards and discriminatory law, serve to reinforce violence against minorities.

### 6.2. Violence against minorities: Part of pre-planned attacks?

Given the frequency and intensity of these attacks, it seems that violent attacks against minorities are part of a larger plan to drive minorities out of the country. Dey (2021) claims that religious fundamentalists, who failed to establish a religiously based country in 1971, are now redoubling their efforts. These fundamentalists actively create communal conflicts and foster religious emotionalism among the general public by disseminating propaganda through various media (Dey, 2021).

Hindus now account for 8.5% - down from 13.5% in 1971- of the entire population of the country (Dey, 2021), with Muslims accounting for 90% of the country's population. If religious minorities face continuous stress and anxiety resulting from such attacks, they may decide to leave the country. Hindus would then no longer be a deciding factor in electoral politics, if their numbers were reduced to 3–4%. In that scenario, religion would then take center stage in the electoral politics of Bangladesh, Dey (2021) asserts.

### 6.3. Increasing fundamentalism

Bangladesh is a sovereign nation with a Muslim majority population. Despite the fact that Islam is listed as the state religion in its constitution, secularism is upheld. Bangladesh's constitution prohibits discrimination based on religion. The state shall ensure equal status and equal rights in the practice of the Hindu, Buddhist, Christian, and other religions, but Islam is the state religion of the Republic, according to the constitution. Any political position cannot support any religion [the constitution states].

Violence, due to religious differences, has a long history in the subcontinent, going back to the late nineteenth century. Religious divisions became a primary cause for the partition of India into India and Pakistan in 1947. Bangladesh (earlier East Pakistan) gained independence in 1971 as a result of conflict when the ethnic Bengali population was humiliated by West Pakistani elites. Since its independence from Pakistan, Bangladesh has prided itself on its secular credentials. Despite the fact that Islam became the state religion of Bangladesh in later years, the constitution also preserves the principle of secularism. Attacks against religious minorities have however continued for decades. Ethirajan (2021) argues that there is a concerted effort in Bangladesh to grab Hindu homes and land, pushing Hindus out of the nation. The ruling Awami League, which has been in office since 2008, has so far not been able to address the rising religious intolerance and extremism. Vast numbers of Hindus have migrated to India, drastically reducing their number in Bangladesh (Ethirajan, 2021). The state's indifference to minority rights is only exacerbating the problem.

### 6.4. Communal violence is a form of violation of human rights

The violence in Bangladesh indicates the growing trend in South Asia toward intolerance of minorities. The religious and ethnic minorities claim that they have been victims of blatant communal politics which cater to the majority communities from Sri Lanka to Pakistan. Bangladesh has seen the formation of local Muslim extremist groups that have been accused of carrying out a wave of killings against the country's secular forces. It has also been exposed to cross-border tensions as a result of India's rising sectarian politics, which has targeted Muslims, particularly those of Bangladeshi heritage (Hasnat and Mashal, 2021).

### 6.5. The lack of penalties for violence against minorities

Religious fundamentalists, particularly members of the former Jamaat-e-Islami, Bangladesh's largest Islamic party, were blamed for attacks on the Hindu population. However, the party has been mostly dormant since several of its leaders were executed for war crimes committed during the 1971 war of independence against Pakistan.

The ruling Awami League party on power has accused religious fundamentalists and promised to take action against the perpetrators. We have also seen from recent cases how the current ruling party activists have planned and executed these attacks (from Nasirnagar, Brahmanbaria, Shalla, Sunamganj, and Chittagong among others). However, little action has been taken since this incidence, and no one has been punished for the attacks. Influential persons can sometimes prevent police from filing case-related to attacks. If the police investigation is delayed, there are high chances of the culprits being discharged without punishment (Welle, 2021).

### 6.6. Lack of legal safeguards

Human rights organizations claim that these attacks are frequently overlooked by the legal system and go unpunished. Thousands of attacks have been registered in the last several years, with the Hindu population usually subjected to violence. According to Ain O Salish Kendra (ASK), a Bangladeshi human rights organization, since 2013 ~3,600 attacks targeting Hindus have occurred in Bangladesh. Over 550 homes and 440 companies were targeted in the attacks, which included vandalism and arson (Ain O Salish Kendro, 2009). According to ASK, there were around 1,670 incidences of vandalism and arson attacks on Hindu temples, idols, and places of worship during that time. In total, 11 Hindus were killed, and 862 others were injured in these attacks. Several cases of sexual assault against Hindu women were also documented. In the absence of proper legal safeguards, the perpetrators of such acts do not feel discouraged, leading to the continuance of violence against minorities.

Minority protection was overlooked in the 1972 Constitution, and only relatively recently, in 2011, was a special provision to protect and develop the “unique local culture and tradition of the tribes, minor races, ethnic sects, and communities” inserted into the Constitution *via* the 15th Constitutional Amendment (Article 23A). Since 1972, there has been an existing provision for the preservation and enhancement of “state language, literature, and the arts”. The term “minorities” however is not defined in the Constitution, despite the fact that Bangladesh, as a member of the international community, is obligated to protect them from all types of discrimination and violence.

### 6.7. Discriminatory law

Most people now use the internet and social media to communicate, to share their thoughts and ideas. As a result, this particular form of media needs to be monitored, and the Information and Communication Technology (ICT) Act was the first piece of legislation in Bangladesh to do so. Passed in 2006, it was later revised in 2013 (Fahad, 2018).

The ICT Act informs that: “If any person deliberately publishes or transmits or causes to be published or transmitted in the website or in electronic form any material which is fake and obscene or whose effect is such as to tend to deprave and corrupt persons who are likely, having regard to all relevant circumstances, to read, see, or hear the matter contained or embodied in it, or causes to deteriorate or create,” they will face punishment (Fahad, 2018). There are however no guidelines in section 57 of the ICT Act to determine specifically what kind of content will harm the “image of the state”, or what would be considered “obscene”. Similarly, “causes to deteriorate or provides a tendency to deteriorate law and orders” is a rather vague definition of an offense, making it impossible for an individual to know what specifically constitutes a criminal offense (Fahad, 2018).

### 6.8. Maintaining community harmony is a joint responsibility

The people and the state, particularly the government and political parties that operate at the local level, have a myriad of responsibilities. Many doubts remain concerning the administration's role in these attacks on minorities. The Prime Minister has repeatedly told ruling party leaders at all levels to strive to keep the area peaceful and orderly. However, on the ground realities say otherwise (Panday, 2021).

A heterogeneous country cannot prosper without integrating disadvantaged groups into politics and society. Peaceful coexistence is the only solution, even if it has to be enforced or coerced by the state. Related to this, Panday (2021) claims that the masterminds of these attacks are motivated by political expediency and financial gain. They are criminals, who are not driven by Hindu or Islamic religious beliefs at all. Others argue that these groups are intentionally targeting minorities in order to put pressure on the government (Panday, 2021).

## 7. Concluding statement

Our examples clearly establish that there is an increasing trend of minority attacks due to misinformation spread on social media platforms. This study shows there is a lack of proper legal mechanisms to address violence against minorities in Bangladesh. The ICT Act, which almost solely covers the Hindu community, should be restrengthened for the protection of all minorities. Based on our findings, we recommend that the Bangladesh government should provide sufficient security for progressive and secular voices in the media against the onslaught of Islamist radicals. It could be done by bringing more stringent rules and regulations, as well as by concentrated efforts at the local levels. The Bangladesh government could establish an education program to raise awareness of religious and ethnic minorities' cultural and religious values among all.

## Data availability statement

The raw data supporting the conclusions of this article will be made available by the authors, without undue reservation.

## Author contributions

All authors listed have made a substantial, direct, and intellectual contribution to the work and approved it for publication.
